# Deep learning-based automatic measurement system for patellar height: a multicenter retrospective study

**DOI:** 10.1186/s13018-024-04809-6

**Published:** 2024-05-31

**Authors:** Zeyu Liu, Jiangjiang Wu, Xu Gao, Zhipeng Qin, Run Tian, Chunsheng Wang

**Affiliations:** 1 Department of Bone and Joint Surgery, The Second Affiliated Hospital of Xi’an Jiaotong University, Xi’an, Shaanxi China; 2 College of Information and Communication Engineering, Harbin Engineering University, Heilongjiang, Harbin China; 3 Department of Orthopedics, Xi’an Honghui Hospital, Xi’an, Shaanxi China; 4 Department of Orthopedics, The Second Affiliated Hospital of Shanxi Medical University, Taiyuan, Shanxi China

**Keywords:** Deep learning, Patellar height index, Radiographic imaging, Keypoint detection

## Abstract

**Background:**

The patellar height index is important; however, the measurement procedures are time-consuming and prone to significant variability among and within observers. We developed a deep learning-based automatic measurement system for the patellar height and evaluated its performance and generalization ability to accurately measure the patellar height index.

**Methods:**

We developed a dataset containing 3,923 lateral knee X-ray images. Notably, all X-ray images were from three tertiary level A hospitals, and 2,341 cases were included in the analysis after screening. By manually labeling key points, the model was trained using the residual network (ResNet) and high-resolution network (HRNet) for human pose estimation architectures to measure the patellar height index. Various data enhancement techniques were used to enhance the robustness of the model. The root mean square error (RMSE), object keypoint similarity (OKS), and percentage of correct keypoint (PCK) metrics were used to evaluate the training results. In addition, we used the intraclass correlation coefficient (ICC) to assess the consistency between manual and automatic measurements.

**Results:**

The HRNet model performed excellently in keypoint detection tasks by comparing different deep learning models. Furthermore, the pose_hrnet_w48 model was particularly outstanding in the RMSE, OKS, and PCK metrics, and the Insall–Salvati index (ISI) automatically calculated by this model was also highly consistent with the manual measurements (intraclass correlation coefficient [ICC], 0.809–0.885). This evidence demonstrates the accuracy and generalizability of this deep learning system in practical applications.

**Conclusion:**

We successfully developed a deep learning-based automatic measurement system for the patellar height. The system demonstrated accuracy comparable to that of experienced radiologists and a strong generalizability across different datasets. It provides an essential tool for assessing and treating knee diseases early and monitoring and rehabilitation after knee surgery. Due to the potential bias in the selection of datasets in this study, different datasets should be examined in the future to optimize the model so that it can be reliably applied in clinical practice.

**Trial registration:**

The study was registered at the Medical Research Registration and Filing Information System (medicalresearch.org.cn) MR-61-23-013065. Date of registration: May 04, 2023 (retrospectively registered).

## Background

The patellar height is important in the anatomy and biomechanics of the patellofemoral joint. Recently, several studies have shown that an abnormal patellar height is associated with various knee diseases. These diseases include patellar dislocation [1, 2], patellar instability [3], Osgood–Schlatter disease [4, 5], anterior knee pain [6], chondromalacia patella [7], and anterior cruciate ligament (ACL) injuries [8, 9]. Moreover, abnormalities in the patellar height are closely linked to complications and poor recovery after total knee arthroplasty (TKA) [10–12], tibial osteotomy [13], and ACL reconstruction [14]. Therefore, early assessment and treatment of abnormal patellar height are vital to effectively control symptoms, prevent and alleviate related diseases, and improve patients’ quality of life.

The patellar height is typically measured directly or indirectly using radiological or magnetic resonance imaging (MRI) methods [15]. However, these standard procedures are lengthy, time-consuming, repetitive, and require additional computational support. They are prone to significant variability among and within observers, which may affect the accuracy of the measurements [16, 17].

Recently, deep-learning algorithms have been increasingly applied across various aspects of the medical field, particularly in orthopedics. Kim et al. [18] automated the detection and segmentation of lumbar vertebrae from radiographs to assess compressive fractures. Similarly, Krogue et al. [19] implemented the automatic identification and classification of hip fractures. Regarding surgical planning, Qu et al. [20] utilized MRI for the precise segmentation of pelvic bone tumors, aiding the development of effective surgical plans for tumor excision and reconstruction. Von Schacky et al. [21] segmented and classified primary bone tumors, whereas Consalvo et al. [22] have detected and differentiated Ewing’s sarcoma and acute osteomyelitis. Leung et al. [23] have predicted the risks of TKA in patients with osteoarthritis for risk assessment. Ye et al. [24] developed a deep learning-based automatic measurement algorithm using a convolutional neural network (CNN) with VGG16 as the encoder. Kwolek et al. [25] employed the YOLO neural network and U-Net for detecting and segmenting the patellofemoral joint, enabling automatic measurements of the Caton-Deschamps index (CDI) and Blackburne-Peel index.

Deep-learning algorithms are particularly effective at navigating the complex anatomical structures and variations between normal and pathological states in humans. They excel at identifying and learning intricate patterns from extensive datasets, which is crucial for accurately measuring patellar height in various radiographic images [26]. Furthermore, the automation capabilities of deep learning significantly streamline the measurement process, reducing both the variability and the time required for manual assessments [27].

Therefore, our objective was to develop a novel deep learning-based algorithm to automatically measure patellar height, to enable high-precision and rapid analysis of medical images.

## Methods

### Study aim, design, and setting

This multicenter retrospective study aimed to develop a deep learning-based algorithm to automatically measure patellar height parameters in lateral knee radiographs and evaluate its performance and generalization ability. We utilized a dataset containing X-ray images from three tertiary level A hospitals.

### Datasets

The images used in this study’s dataset were obtained from three tertiary A-grade comprehensive hospitals: The Second Affiliated Hospital of Xi’an Jiaotong University, Xi’an Honghui Hospital, and The Second Affiliated Hospital of Shanxi Medical University. This retrospective study was approved by the ethics committees of the three hospitals. The ethics committees waived the requirement for informed consent due to the study’s retrospective nature. We continuously collected imaging and clinical data between April 2022 and December 2023 through the imaging and hospital information system. Patients aged ≥ 20 years (with mature bones) undergoing a lateral knee radiograph were eligible for inclusion. The exclusion criteria were as follows: (1) knee osteoarthritis (Kellgren–Lawrence grade > 2); (2) poor quality radiographs with insufficient rotation (the distance between the edge of the femoral condyles ˃5 mm) or knee flexion ˂30°; (3) overlapping knees; and (4) unclear superior or inferior poles of the patellar or patellar ligament endpoints. The osteoarthritis criteria were used to include radiographs with clear patellar height markers. We retrospectively analyzed 3,923 knee joints, of which 2,341 met the inclusion criteria. A random selection of 90% of the cases from The Second Affiliated Hospital of Xi’an Jiaotong University (2,017 X-ray images) was used for training, and 10% (224 X-ray images) were used as the internal test set. For external validation, we further evaluated datasets from the Xi’an Honghui Hospital (60 X-ray images as external test set 1) and The Second Affiliated Hospital of Shanxi Medical University (40 X-ray images as external test set 2).

**Fig. 1:**
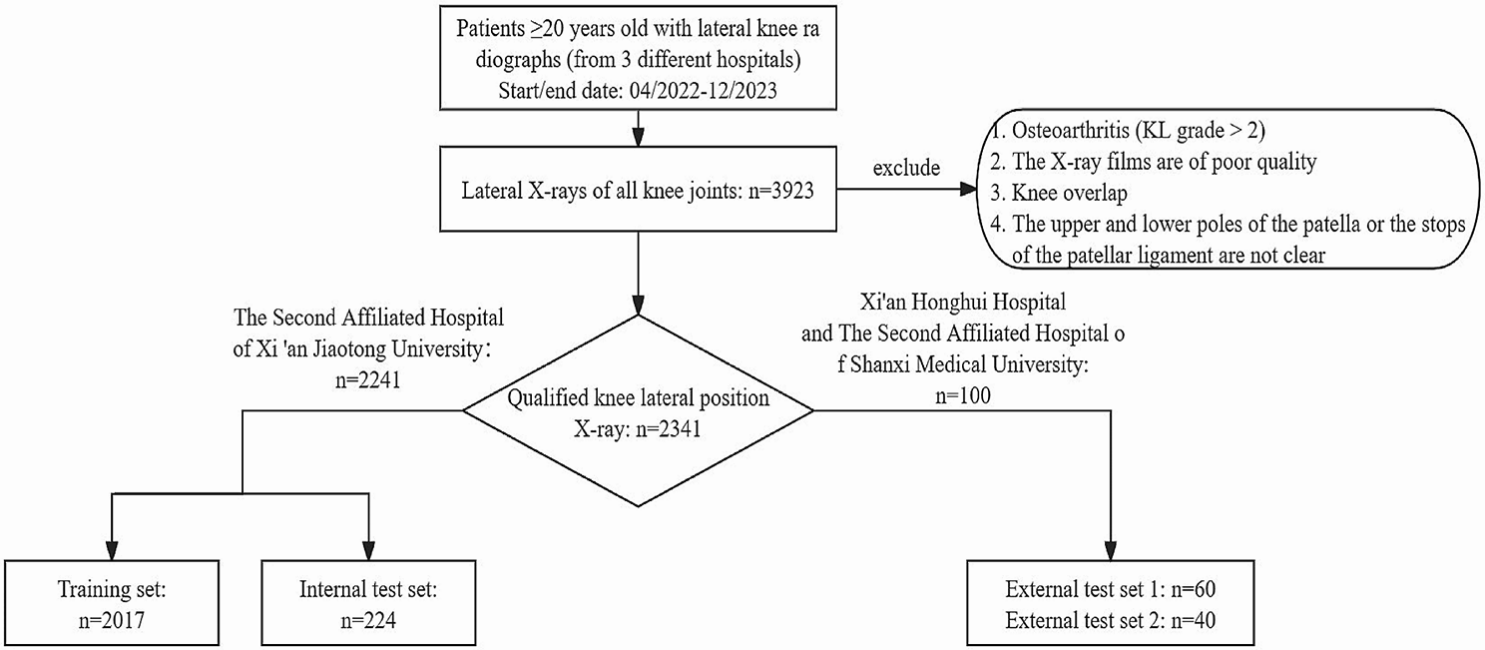
Data collection process

**Fig. 2:**
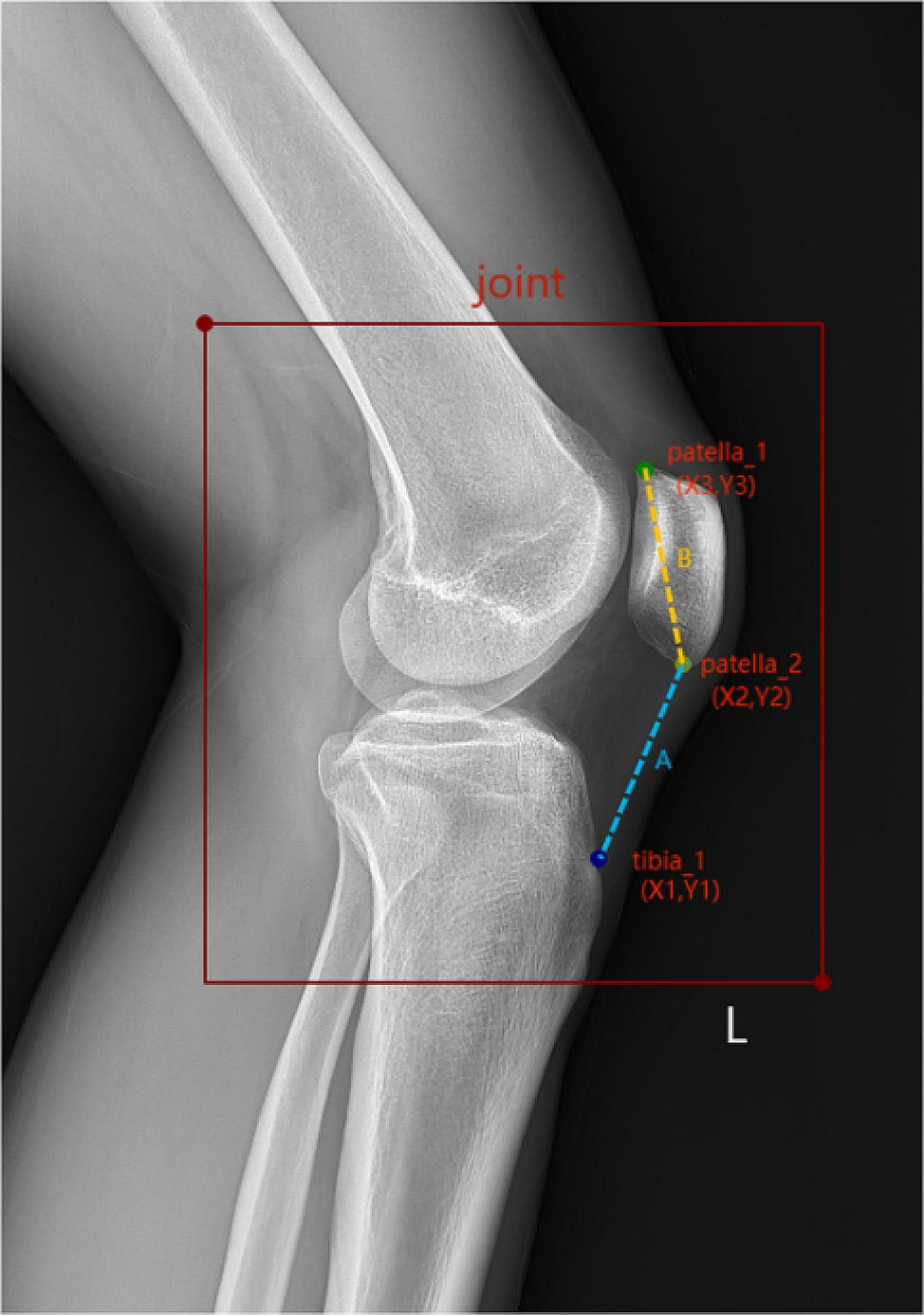
Keypoint marking and calculation method for the ISI of PH. Patella_1, upper pole of patella; patella_2, inferior pole of patella; tibia_1, insertion of the patellar ligament. The ISI is the ratio of A to B; ${\rm{A}}\, = \,\sqrt {{\rm{X}}1 - {\rm{X}}2{^2} + {\rm{Y}}1 - {\rm{Y}}2{^2}}$, ${\rm{B}}\, = \,\sqrt {{\rm{X}}2 - {\rm{X}}3{^2} + {\rm{Y}}2 - {\rm{Y}}3{^2}}$ ISI, Insall–Salvati Index

**Fig. 3:**
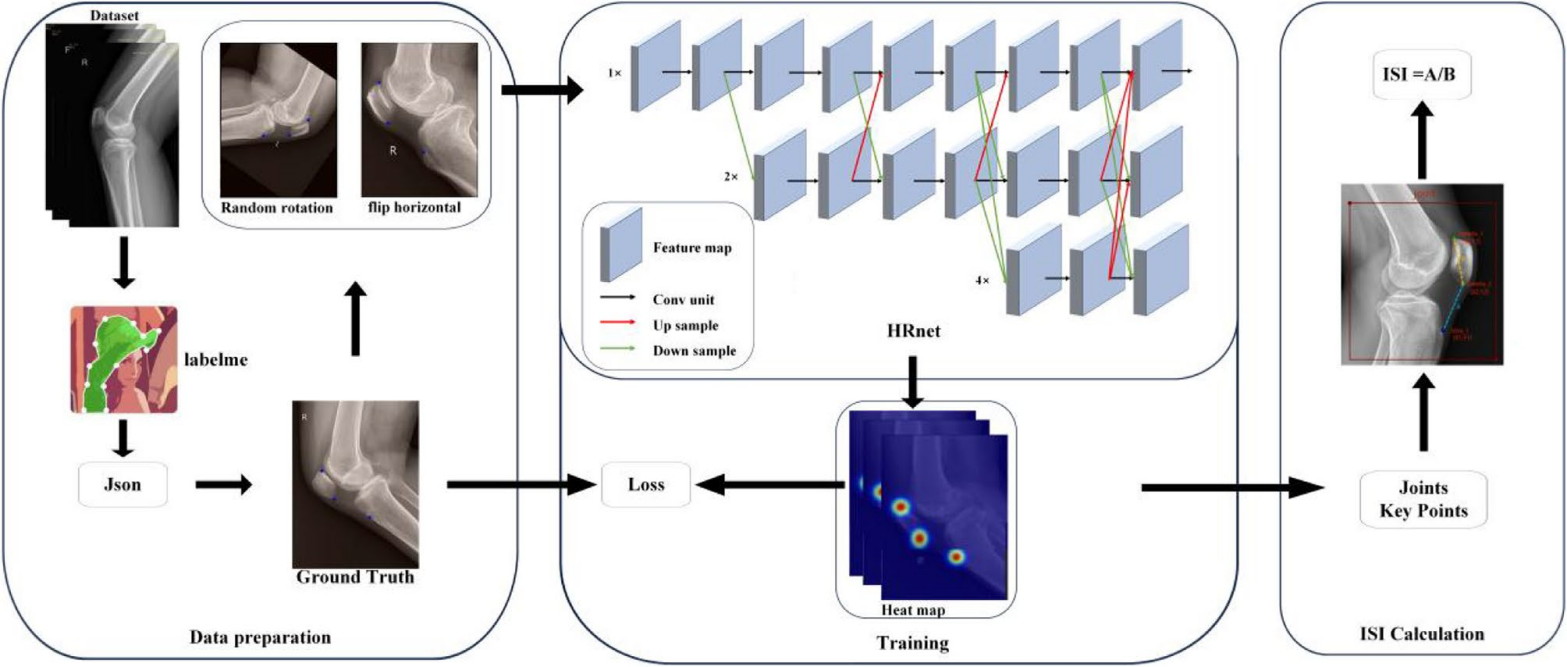
Overall model architecture diagram

The dataset required labeling before training the neural network. The LabelMe software was used to manually annotate the keypoints in the lateral knee radiographs, generating JSON format annotation files. Three keypoints were labeled from the 2,341 images. Images from each of the three hospitals were annotated by radiologists (R1, R2, and R3) with over 10 years of experience from their respective institutions. Figures 1, 2 and 3 illustrate the data collection process, keypoint annotation demonstration, and overall model architecture, respectively.

### Dataset preparation

An experiment was conducted using the PyTorch 1.10 framework in Python 3.8 (Python Software Foundation, Wilmington, DE, USA) and Cuda 11.3 (Nvidia Corp., Santa Clara, CA, USA) environments to demonstrate the performance and effectiveness of the constructed model. The experimental setup was based on the Ubuntu 18.04 operating system (Canonical Ltd., London, England) with an Intel i7-10700 F processor (Intel, Santa Clara, CA, USA) and was equipped with an Nvidia GeForce RTX 4070 GPU (Nvidia Corp.) and 32 GB RAM. We optimized several parameters of our deep learning models to balance computational efficiency and training effectiveness based on the capabilities of our NVIDIA GeForce RTX 4070 GPU, which has 12 GB of VRAM. We chose a batch size of 8 after extensive testing, which maximized VRAM usage and model training speed, particularly when using the computationally intensive pose_hrnet_w48 model with an input size of 384 × 288. For consistency, this batch size was applied across all models. The Adam optimizer was selected for its robust performance and rapid convergence. To manage computational load while preserving image quality, we experimented with input sizes of 256 × 192 and 384 × 288 pixels. Through preliminary testing, this approach not only reduced the volume of data processed by the model but also enhanced training and inference efficiency without significant feature loss. Therefore, we uniformly adjusted the image size to 256 × 192 or 384 × 288 pixels and employed data augmentation techniques such as random rotation, scaling, and flipping to enhance the model’s robustness and generalization ability.

### Model selection

The residual network for human pose estimation (Pose_ResNet) is an advanced human pose estimation method that leverages the robustness of deep residual networks (ResNets) to effectively locate human body keypoints [28, 29]. The network initially extracts image features through convolutional layers, which can recognize complex patterns in the images and provide the necessary visual information for detecting human-body keypoints. The output is a set of heat maps, each corresponding to a specific body keypoint, representing the probabilistic distribution of the keypoint’s possible locations within the image. During training, the loss function typically compared the predicted heat maps with the true heat maps to optimize the network parameters. The final keypoint locations are determined through post-processing steps such as finding the local maxima in the heatmaps. This architecture was built on the fundamental principles of ResNet by integrating deep convolutional layers with residual connections. These connections mitigate the issue of vanishing gradients, thereby enabling the training of deeper networks to enhance feature extraction without losing crucial information during the process.

The High-Resolution Network for Human Pose Estimation (Pose_HRNet) is an efficient network for human pose estimation designed to improve keypoint detection accuracy by maintaining high-resolution feature maps throughout the process [30–33]. Starting with a high-resolution subnetwork, it progressively adds high-to-low-resolution subnetworks in more stages. Then, it connects multiresolution subnetworks in parallel to learn features on different scales. This multiscale approach enhances the robustness of the model to various challenges in pose estimation, such as occlusions and varying sizes. The global context and local detail information are effectively fused throughout the process by exchanging information across parallel multiresolution subnetworks. Finally, the keypoints are estimated on the network’s output high-resolution feature maps. This approach benefits from semantically richer and spatially precise result representations.

### Performance and generalization of keypoint detection

We assessed the landmark detection performance of five models, pose_resnet_50, pose_resnet_101, pose_resnet_152, pose_hrnet_w32, and pose_hrnet_w48, using root mean square error (RMSE), object keypoint similarity (OKS), and percentage of correct keypoints (PCK). Furthermore, we compared the parameter counts and computational complexities of the models. Subsequently, an appropriate model was selected to evaluate the performance of the external test dataset. This was performed to assess the accuracy of knee joint radiographs obtained from different devices across various algorithms.

- RMSE measures the model accuracy using the square difference between the average predicted and actual values, indicating the error magnitude.
- OKS evaluates keypoint detection accuracy by considering object scale and keypoint distance, offering a normalized accuracy measure that penalizes larger errors more.
- PCK calculates the share of keypoints accurately detected within a set distance from true positions, reflecting the model’s precision in keypoint localization.

### Performance and generalization of insall–Salvati index (ISI) measurement

The analyses compared the average ISI test results across the three test sets. In addition, we calculated the intraclass correlation coefficient (ICC) to evaluate the reliability and consistency of the manual and automatic measurements. The ICC reflects abstract consistency, with values ≥ 0.75 considered sufficiently reliable [34]. This approach was used to assess the performance and generalizability of ISI measurements.

### Statistical analysis

Statistical analyses were performed using PASW Statistics v18 (IBM Corp., Armonk, NY, USA) and Microsoft Excel 2019 (Microsoft Corp., Redmond, WA, USA). We employed ANOVA to compare the mean ages of different groups to identify any statistically significant differences in age distributions. For categorical variables such as sex, side, osteoarthritis grade, and surgery status, chi-square tests were utilized to assess the homogeneity of these variables across the groups. All statistical tests were two-tailed, and a p-value of less than 0.05 was considered to indicate statistical significance.

## Results

### General data distributions

In this study of 2,241 knee radiographs, the average ages of the patients forming the training, internal, first external, and second external sets, were 50.10 ± 17.77 years (range, 20–87 years), 50.76 ± 16.96 years (range, 21–83 years), 48.88 ± 15.01 years (range, 23–86 years), and 47.43 ± 13.01 years (range, 20–81 years), respectively. Table 1 presents the overall patient characteristics.

**Table 1:** Patient characteristics

		Training data set, *n* (%)	Internal test set, *n* (%)	External test set, *n* (%)		*P*-value
				External test set 1	External test set 2	
No. of images		2017	224	60	40	
Age* (years)		50.10 ± 17.77	50.76 ± 16.9	53.88 ± 15.01	49.43 ± 13.01	0.642
Sex						0.161
	Male	904(45)	103(46)	25(42)	11(28)	
	Female	1113(55)	121(54)	35(58)	29(73)	
Side						0.801
	Left	1060(53)	123(55)	29(48)	22(55)	
	Right	957(47)	101(45)	31(52)	18(45)	
Osteoarthritis						0.001
	Grade 0	1410(70)	150(67)	40(67)	20(50)	
	Grade 1	365(18)	39(17)	15(25)	6(15)	
	Grade 2	242(12)	35(16)	5(8)	14(35)	
Surgery						0.586
	Preoperatively	1810(90)	196(88)	54(90)	34(85)	
	Postoperatively	207(10)	28(13)	6(10)	6(15)	

* Data are presented as mean ± standard deviation

Statistical analysis indicated significant differences in the distribution of osteoarthritis severity across the datasets (*p* < 0.05)

### Performance and generalization of keypoint detection

#### Quantitative analysis

**Fig. 4:**
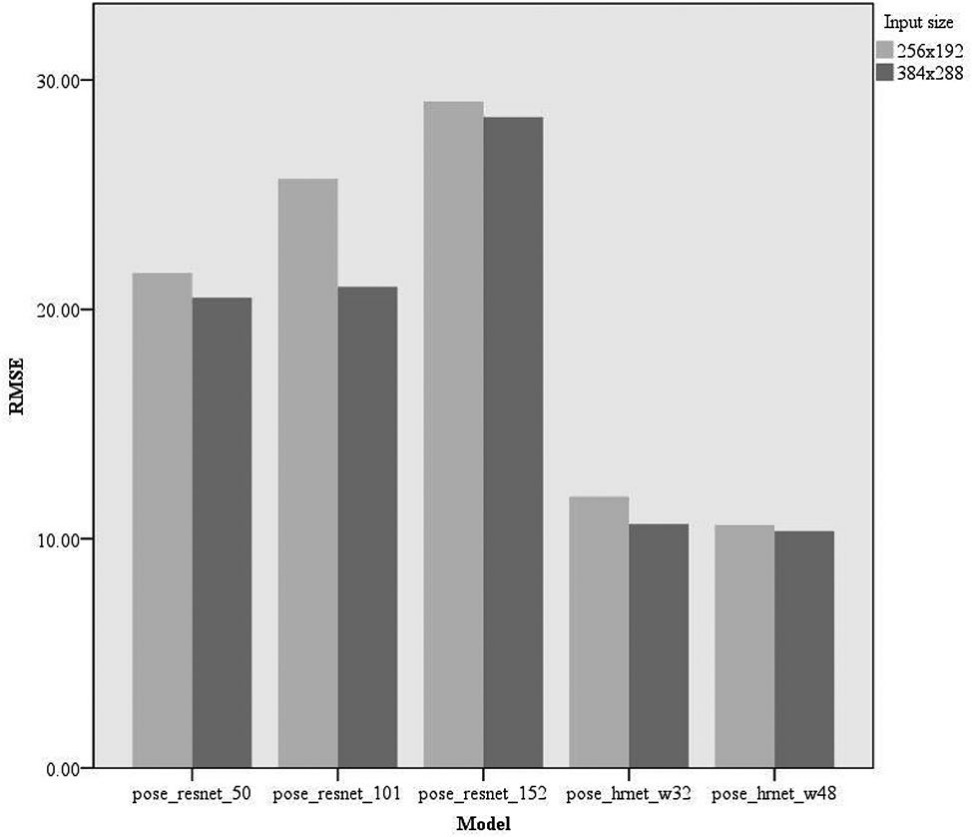
Comparison of RMSE of different models RMSE, root mean square error

**Fig. 5:**
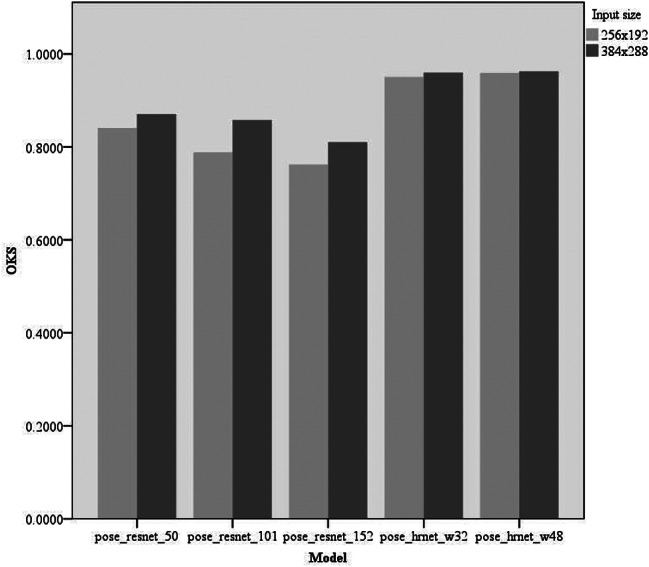
Comparison of OKS of different models OKS, object keypoint similarity

**Fig. 6:**
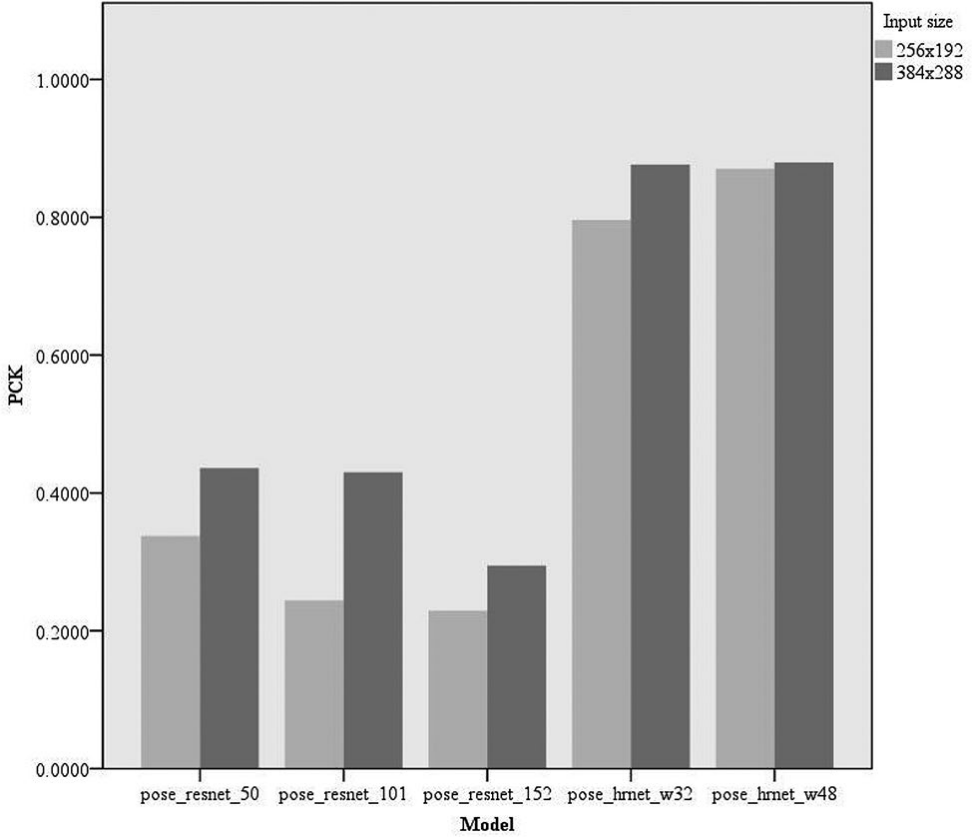
Comparison of PCK of different models PCK, percentage of correct keypoints

Experiments were conducted on keypoint-detection tasks using different deep-learning models. We primarily compared five models: pose_resnet_50, pose_resnet_101, pose_resnet_152, pose_hrnet_w32, and pose_hrnet_w48. We examined the impact of the input sizes of 256 × 192 and 384 × 288 pixels on model performance. In the comparative analysis, we focused on the number of model parameters (#Params), computational complexity (GFLOPs), and three performance metrics: RMSE, OKS, and PCK. The results are shown in Table 2; Figs. 4, 5 and 6.

Model Parameters and Computational Complexity: The parameter counts for pose_resnet_50, pose_resnet_101, and pose_resnet_152 were 34.0 M, 53.0 M, and 68.6 M, respectively, with computational complexities of 8.9, 12.4, and 15.7 GFLOPs, respectively. In contrast, pose_hrnet_w32 and pose_hrnet_w48 had parameter counts of 28.5 M and 63.6 M, respectively, with computational complexities of 7.1 and 14.6 GFLOPs, respectively. A positive correlation was observed between the model parameter count and computational complexity. The results of the performance analysis are as follows:

- RMSE: The pose_hrnet_w48 model scored 10.60 and the pose_hrnet_w32 score was 11.84. However, the pose_resnet series had higher RMSE values with pose_resnet_152 having the highest value of 29.06.
- OKS: The pose_hrnet_w48 model scored the highest at 0.9592, while pose_hrnet_w32 scored 0.9505. In the pose_resnet series, pose_resnet_50 scored 0.8408.
- PCK: The pose_hrnet_w48 model had the highest score of 0.8705. The pose_hrnet_w32 model scored 0.7961. The Pose_ResNet series generally exhibited lower PCK scores.

**Table 2:** Performance metrics for keypoint detection on the internal test set

Model	Input size	#Params	GFLOPs	FPS	RMSE	OKS	PCK
Pose_resnet_50	256 × 192	34.0 M	8.9	162.12	21.58	0.8408	0.3378
Pose_resnet_101	256 × 192	53.0 M	12.4	85.81	25.69	0.7884	0.2440
Pose_resnet_152	256 × 192	68.6 M	15.7	63.42	29.06	0.7620	0.2292
Pose_hrnet_w32	256 × 192	28.5 M	7.1	33.05	11.84	0.9505	0.7961
Pose_hrnet_w48	256 × 192	63.6 M	14.6	31.20	10.60	0.9592	0.8705
Pose_resnet_50	384 × 288	34.0 M	20.0	149.26	20.51	0.8705	0.4360
Pose_resnet_101	384 × 288	53.0 M	27.9	87.44	20.98	0.8577	0.4301
Pose_resnet_152	384 × 288	68.6 M	35.3	60.99	28.38	0.8101	0.2946
Pose_hrnet_w32	384 × 288	28.5 M	16.0	32.96	10.64	0.9598	0.8765
Pose_hrnet_w48	384 × 288	63.6 M	32.9	31.80	10.33	0.9625	0.8795

Model: Indicates the model used. Input size: Size of input to the network. #Params: The amount of parameters in the model. GFLOPs: demand for computational capability. FPS: number of frames processed by the image per second. RMSE: root mean square error used to measure the model’s accuracy. OKS: object keypoint similarity that reflects the accuracy of keypoint detection. PCK: percentage of correct keypoints, another metric for assessing keypoint detection performance

### Qualitative analysis

**Fig. 7:**
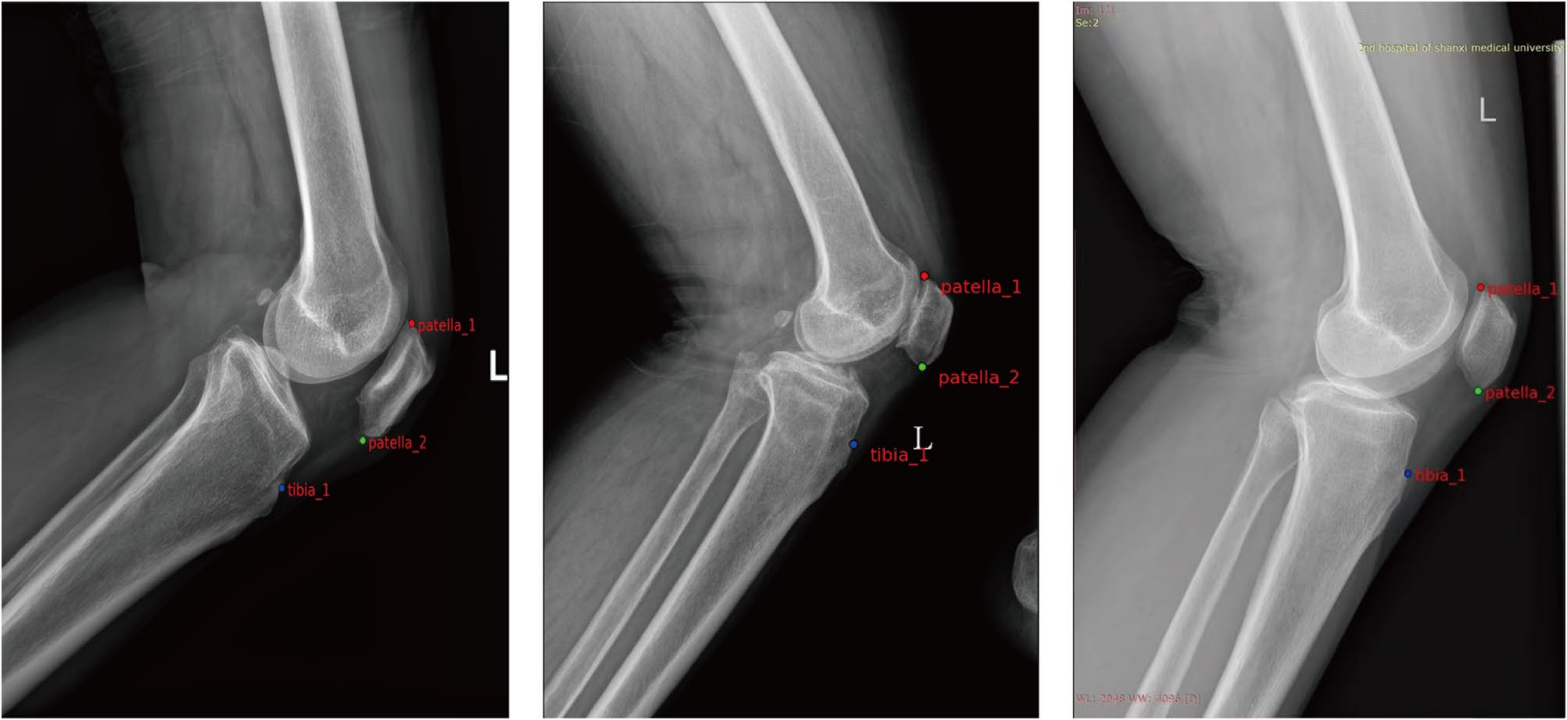
Visualized results of keypoint detection on three datasets. From left to right are the internal test set, external test set 1, and external test set 2

**Fig. 8:**
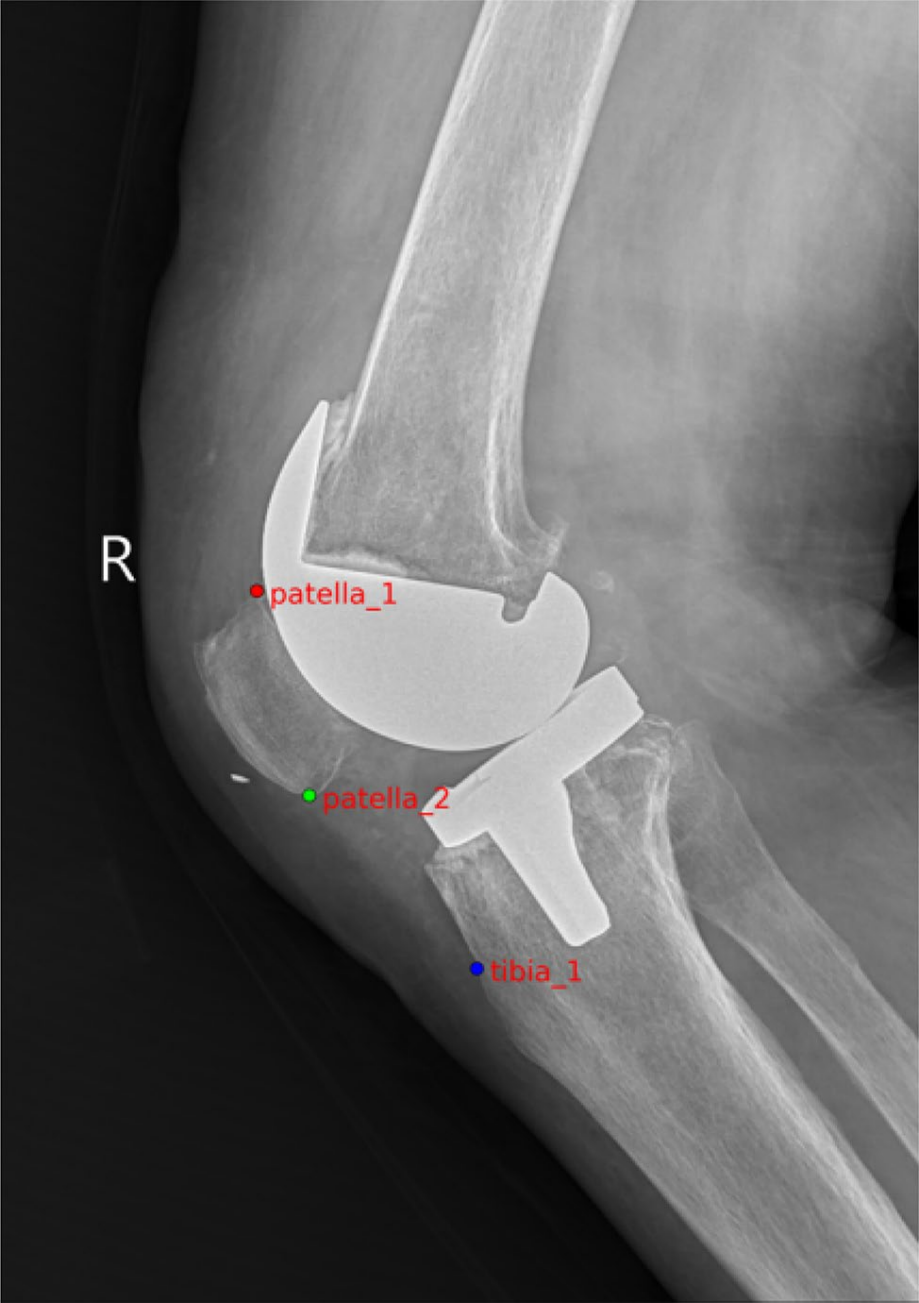
Rendering of keypoint detection after total knee replacement

From the image analysis perspective, our patellar height measurement system can accurately identify the patella’s upper and lower poles and the patellar tendon’s endpoints. The precision of this identification is high, with virtually no deviation discernible to the human eye (Fig. 7). Therefore, it is evident from the effectiveness diagrams of patellar keypoint detection after total knee arthroplasty that the patellar height measurement system can still effectively identify and measure keypoints even with the implantation of knee prostheses (Fig. 8).

### Generalized analysis

Our study also included an analysis of the model’s generalizability. Tables 3 and 4 show the model’s performance on external test sets from other institutions. In the external test set 1 from Xi’an Honghui Hospital, as presented in Table 3, the HRNet model exhibited an RMSE value between 2 and 3, an OKS > 0.96, and a PCK > 0.99. Similarly, on external test set 2 from the Second Affiliated Hospital of Shanxi Medical University, as presented in Table 4, HRNet’s RMSE ranged between 7 and 9, with an OKS > 0.94 and a PCK > 0.88.

**Table 3:** Performance metrics for keypoint detection on external test set 1

Model	Input size	#Params	GFLOPs	FPS	RMSE	OKS	PCK
Pose_hrnet_w32	256 × 192	28.5 M	7.1	33.14	2.4566	0.9674	1.0000
Pose_hrnet_w48	256 × 192	63.6 M	14.6	31.84	2.3717	0.9665	0.9944
Pose_hrnet_w32	384 × 288	28.5 M	16.0	32.99	2.1979	0.9703	0.9944
Pose_hrnet_w48	384 × 288	63.6 M	32.9	30.48	2.0737	0.9747	1.0000

**Table 4:** Performance metrics for keypoint detection on external test set 2

Model	Input size	#Params	GFLOPs	FPS	RMSE	OKS	PCK
Pose_hrnet_w32	256 × 192	28.5 M	7.1	30.49	8.7414	0.9473	0.8917
Pose_hrnet_w48	256 × 192	63.6 M	14.6	31.18	8.1060	0.9494	0.8833
Pose_hrnet_w32	384 × 288	28.5 M	16.0	32.43	7.1375	0.9571	0.9000
Pose_hrnet_w48	384 × 288	63.6 M	32.9	31.74	8.0596	0.9544	0.9000

### Performance and generalization of the ISI measurement

Radiologists with over 10 years of experience calculated the ISI index values with averages of 1.0444 ± 0.1453, 1.0587 ± 0.1727, and 1.0826 ± 0.1448 for the internal test set, external test set 1, and external test set 2, respectively. The deep learning algorithm based on pose_hrnet_w48 automatically calculated ISI index values with averages of 1.0377 ± 0.1323, 1.0637 ± 0.1900, and 1.0492 ± 0.1363 for the internal test set, external test set 1, and external test set 2, respectively (Table 5). ICC was used to evaluate the consistency between manual and automatic measurements. For R1 vs. Model, the ICC = 0.809; R2 vs. Model: ICC = 0.885; and R3 vs. Model: ICC = 0.830 (Table 6).

**Table 5:** Comparison of average ISI test results on three test sets

Date set	Ground truth	Predicted value
Internal data set	1.0444 ± 0.1453	1.0377 ± 0.1323
External data set 1	1.0587 ± 0.1727	1.0637 ± 0.1900
External data set 2	1.0826 ± 0.1448	1.0492 ± 0.1363

Ground truth: Patellar height indices calculated by the radiologists with over 10 years of experience. Predicted value: Patellar height indices automatically calculated using the deep learning algorithm pose_hrnet_w48. The results of each index are presented as mean ± standard deviation

**Table 6:** Evaluation of the patellar height measurement system using ICC

	ICC
R1 vs. Model	0.809
R2 vs. Model	0.885
R3 vs. Model	0.830

R1: Radiologist from the Second Affiliated Hospital of Xi’an Jiaotong University; R2: Radiologist from Xi’an Honghui Hospital; R3: Radiologist from the Second Affiliated Hospital of Shanxi Medical University; Model: A deep-learning algorithm model based on pose_hrnet_w48; ICC: Intraclass Correlation Coefficient

## Discussion

Currently, insufficient attention has been given to the measurement of patellar height indices, primarily for the following reasons. First, there is a lack of comprehensive understanding among physicians regarding the role of this index in the diagnosis and assessment of knee joint diseases. However, the patellar height index is crucial to diagnosing abnormal patellar positions and related diseases. Balcarek et al. [2] confirmed that patella alta significantly correlated with increased incidences of patellar dislocations, identifying it as a key risk factor for instability. Dejour et al. [3] found that 24% of patients with patellar instability exhibited patella alta, which impaired knee mechanics and heightened dislocation risks. Similarly, Visuri et al. [5] linked patella alta with Osgood–Schlatter disease, emphasizing its typical presentation of a higher patellar position. Luyckx et al. [6] explored how patella alta affected anterior knee pain by altering joint forces and pressures. Lu et al. [7] reported that abnormal patellar heights disrupted knee biomechanics, potentially increasing cartilage damage. Furthermore, Degnan et al. [9] discovered that a high Insall–Salvati ratio was a significant indicator of ACL damage risks in children, while Nishizawa et al. [12] noted that patella elevation in posterior-stabilized TKA led to increased joint space by reducing flexion restrictions.

Second, the manual measurement of patellar height indices involves subjectivity and variability, as doctors or technicians rely on personal experience and judgment, leading to potentially different results among various people taking measurements [16, 17]. Finally, manual measurements are time-consuming and inefficient, particularly when dealing with large datasets. Therefore, there is an urgent need to develop an automated, objective, and rapid tool to measure patellar height indices.

With the widespread application of deep learning algorithms in the medical field, researchers have begun to explore using this advanced technology to automatically measure the patellar height index. Ye et al. [24] developed an automatic measurement algorithm based on deep learning, employing a CNN framework for landmark detection, with the VGG16 network serving as the core encoder. This method demonstrated high accuracy for the ISI, CDI, and a new method proposed by the Keerati Index (KI). However, it was slightly less accurate for the modified CDI. Kwolek et al. [25] used the “You Only Look Once” neural network to detect the patellofemoral joint area. They employed U-Net for bone segmentation, enabling the automatic measurement of the CDI and Blackburne–Peel index, thereby facilitating the calculation of the patellar height.

This study introduces a novel measurement system of patella height based on deep learning that integrates an HRNet for two-dimensional keypoint detection and mathematical formulae for analyzing joint coordinates, requiring only a single image input to obtain results. To ensure the reliability of the labeled data for the patellar height measurement system, several steps were taken to address inter-observer variability and to enhance consistency during the annotation process. First, the key points on the lateral knee radiographs were manually annotated by experienced radiologists, each with over ten years of experience at their respective institutions. This level of expertise ensured high standards of annotation accuracy. Additionally, the annotations underwent multiple review rounds to ensure no significant discrepancies between observers. Second, the ICC was used to evaluate the consistency between manual and automatic measurements, with ICC values ranging from 0.809 to 0.885. The close ICC values indirectly indicate a high reliability in annotations among different observers, as the model’s automatic measurements were trained using the manual annotations from one hospital’s radiologists. Thirdly, the LabelMe software was used to label the dataset, ensuring standardized annotations across all images. These measures collectively helped to maintain the reliability of the annotations, enabling the effective training and validation of the deep learning models for measuring patellar height.

All hardware selections were made to facilitate the training and inference of our models. We first considered software environment compatibility; PyTorch 1.10 and Python 3.8 are compatible with the reproduction code of our models. The choice of CUDA version 11.3 was dictated by its compatibility with the selected RTX 4070 GPU and the PyTorch framework. Ubuntu 18.04, a popular and stable Linux distribution, was chosen for its reliability and security, although the version of the operating system does not impact experimental results. In terms of hardware performance, the choice of GPU is crucial. Ideally, the more advanced the GPU, the better; however, given our current experimental conditions, we used the Nvidia GeForce RTX 4070 GPU. It has 12 GB of VRAM and 5888 CUDA cores, offering 29 TFLOPS of computational power, more than sufficient for training HRNet. The Intel i7-10700 F processor, with 8 cores and 16 threads, a base frequency of 2.9 GHz, and a turbo frequency of 4.8 GHz, provides robust multi-core and high-frequency capabilities suitable for CPU-intensive tasks such as data preprocessing and model evaluation. The 32 GB of memory ensures that training is not bottlenecked by memory limitations. Overall, these choices were made based on a comprehensive consideration of experimental conditions, compatibility, and performance.

The differences in the distribution of osteoarthritis severity across the datasets (*p* < 0.05) may be linked to the smaller sample sizes in the external test sets. However, due to the large sample size of the training set, it may not affect the effectiveness of key point identification in the test set.

Lower RMSE values correlate with higher model accuracy in predicting patellar height, crucial for ensuring reliable measurements in clinical environments. Higher OKS scores signify that the model’s predictions are both precise and consistent across various knee sizes and positions, enhancing its utility in diverse clinical scenarios. Additionally, a high PCK score demonstrates the model’s reliability in identifying keypoints on the patella and related structures, essential for diagnosing conditions such as patellar instability or dislocation. Pose_hrnet_w48 and pose_hrnet_w32 demonstrated higher accuracy and efficiency than other models in this keypoint detection task, especially pose_hrnet_w48, which performed the best across all performance metrics (Figs. 4, 5 and 6). Although the pose_hrnet series models had higher parameter counts and computational complexities, their performance improvements were significant. Conversely, although the pose_resnet series models had relatively lower computational complexities, their accuracy and efficiency in keypoint detection need to be enhanced. Therefore, selecting an appropriate model for keypoint detection tasks requires a comprehensive consideration of the availability of computational resources and the demand for detection performance. The speed of the various models discussed in this study is sufficient to meet the needs of clinical real-time image processing. Given the critical importance of accuracy in patellar height measurement in clinical practice, we ultimately selected the pose_hrnet_w48 model from HRNet as the benchmark model for our measurement system. Despite its larger parameter size and relatively high computational demand (measured in GFLOPS), it excelled in key performance metrics, achieving an OKS of 0.9625, a RMSE of 10.33, and a PCK of 0.8795. Compared with models based on ResNet, this represents a significant improvement in these metrics. Furthermore, the pose_hrnet_w48 model processes images at 31.80 frames per second (FPS), indicating its capacity to handle 31.80 images per second.

The model’s performance on external test sets further showcased the strong generalizability of the HRNet model for detecting knee joint keypoints across various datasets. In addition, a comparison of the average ISI across the three test sets shows that the difference between the predicted and actual ISI values is negligible. A comparison of the ISI values obtained by radiologists from different hospitals (R1, R2, R3) with those calculated by our deep learning model based on pose_hrnet_w48 for patellar height measurement showed excellent reliability in the results obtained by the artificial intelligence calculation compared with those by the radiologists, indicating that the model has produced results similar to those of radiologists with extensive experience (R1 vs. Model: ICC = 0.809; R2 vs. Model: ICC = 0.885; R3 vs. Model: ICC = 0.830). These results demonstrate the precision and reliability of the patellar height measurement system based on pose_hrnet_w48 across various datasets.

This system’s primary advantages and improvements are mainly reflected in the following aspects. First, unlike the serial convolution method from high to low resolution adopted by existing networks such as ResNet and vanishing gradient (VGGNet), HRNet employs a parallel connection strategy. This maintains a high-resolution expression throughout the inference process and achieves multiscale fusion, allowing for a richer and more accurate estimation of keypoints on high-resolution feature maps [30]. Second, considering that abnormalities in patellar height are closely associated with complications or poor recovery after TKA [10–12] and ACL reconstruction [14], we specifically included the lateral knee radiographs of patients after these surgeries in our custom dataset. Finally, the training dataset for this study was exclusively derived from the Second Affiliated Hospital of Xi’an Jiaotong University, while datasets from the Second Affiliated Hospital of Shanxi Medical University and the Xi’an Honghui Cross Hospital were used solely for testing to validate the generalization capabilities of the deep learning model. Differences in imaging protocols and parameter settings across hospitals are real, but were substantially mitigated during the image preprocessing stage of the deep learning model through adjustments in resolution and the application of data augmentation techniques such as random flipping and rotation. These methods significantly enhanced the model’s robustness. The rationale behind this setup was to test the applicability of the model trained on data from the Second Affiliated Hospital of Xi’an Jiaotong University in broader contexts. The successful performance of the model on datasets from various hospitals indicates its strong potential for clinical application.

In the future, the application of automated patellar height measurement will reduce human errors and variability associated with manual methods, which is critical for diagnosing diseases related to abnormal patellar heights. Accurate patellar height indices will also aid orthopedic surgeons in devising treatment strategies, including surgical planning such as alignment and balance of the patellofemoral joint in TKA. Additionally, this technology can effectively monitor disease progression and recovery post-surgery, ensuring timely and appropriate interventions that can shorten recovery times and improve overall patient outcomes.

Compared to previously developed models for automatically measuring patellar height using deep learning, this study utilizes a large, multicenter dataset and the advanced HRNet architecture, achieving notably low RMSE and high OKS and PCK scores. Despite the outstanding results achieved in this study, there were some limitations. First, training and testing the model on specific hospital datasets may not fully represent the global diversity of knee joint anatomy. Additionally, county and township hospitals may employ different imaging protocols for knee X-rays, which could impact the model’s ability to accurately detect keypoints. Second, the dataset primarily included images with clear patellar height markers, which may have affected the outcomes in patients with abnormal patellar morphology. Third, as a purely linear measurement method, the ISI only considers the patellar height parameter and fails to comprehensively evaluate other anatomical patella structures, such as the patellar type and posterior tibial slope. To mitigate these limitations, future research should train the model with datasets from different races, regions, populations, and devices and focus on optimizing specific patient groups to enhance the model’s applicability across different patient types. By integrating more closely with clinical practice, the model could be guided towards improvements and optimizations for patients with abnormal patellar morphology. Furthermore, consideration should be given to introducing more anatomical structure indicators to comprehensively assess patellar anatomy and the use of three-dimensional imaging techniques (such as computed tomography and MRI) for a comprehensive assessment of patellar anatomy and function. By implementing these measures, we anticipate effectively improving the limitations of the existing model, thereby enabling its true application in clinical settings.

## Conclusions

This study successfully developed and validated a deep learning-based automatic patellar height measurement system. This system can accurately measure the patellar height index, performing on par with experienced radiologists. Extensive dataset testing demonstrated the system’s excellent generalization ability and reliability, particularly for processing radiographs from different hospitals and equipment. This measurement system is expected to help in the assessment, treatment, and postoperative monitoring of knee joint diseases, thereby providing a powerful tool for enhancing patients’ quality of life. Due to the potential bias in the selection of datasets in this study, the model still has some shortcomings. In the future, further optimizing and incorporating more anatomical structure indicators will significantly improve the application scope and accuracy of the system, offering a more precise and comprehensive assessment tool for clinical use.

## Data Availability

The datasets used and/or analyzed in the current study are available from the corresponding author upon reasonable request.

## Abbreviations

RMSE: Root mean square error
OKS: Object keypoint similarity
PCK: Percentage of correct keypoints
ICC: Intraclass correlation coefficient
ResNet: Residual network for human pose estimation
HRNet: High-resolution network for human pose estimation
MRI: Magnetic resonance imaging
TKA: Total knee arthroplasty
ACL: Anterior cruciate ligament
ISI: Insall–Salvati index
CNN: Convolutional neural network
VGG16: Visual geometry group 16
CDI: Caton–Deschamps index
KI: Keerati index

## References

[1] Jaquith BP, Parikh SN (2017). Predictors of recurrent patellar instability in children and adolescents after first-time dislocation. J Pediatr Orthop.

[2] Balcarek P, Ammon J, Frosch S, Walde TA, Schüttrumpf JP, Ferlemann KG (2010). Magnetic resonance imaging characteristics of the medial patellofemoral ligament lesion in acute lateral patellar dislocations considering trochlear dysplasia, Patella alta, and tibial tuberosity-trochlear groove distance. Arthroscopy.

[3] Dejour H, Walch G, Nove-Josserand L, Guier C (1994). Factors of patellar instability: an anatomic radiographic study. Knee Surg Sports Traumatol Arthrosc.

[4] Aparicio G, Abril JC, Calvo E, Alvarez L (1997). Radiologic study of patellar height in Osgood-Schlatter disease. J Pediatr Orthop.

[5] Visuri T, Pihlajamäki HK, Mattila VM, Kiuru M (2007). Elongated patellae at the final stage of Osgood-Schlatter disease: a radiographic study. Knee.

[6] Luyckx T, Didden K, Vandenneucker H, Labey L, Innocenti B, Bellemans J (2009). Is there a biomechanical explanation for anterior knee pain in patients with Patella alta? Influence of patellar height on patellofemoral contact force, contact area and contact pressure. J Bone Joint Surg Br.

[7] Lu W, Yang J, Chen S, Zhu Y, Zhu C (2016). Abnormal patella height based on Insall-Salvati ratio and its correlation with patellar cartilage lesions: an extremity-dedicated low-field magnetic resonance imaging analysis of 1703 Chinese cases. Scand J Surg.

[8] Akgün AS, Agirman M (2021). Associations between anterior cruciate ligament injuries and Patella alta and trochlear dysplasia in adults using magnetic resonance imaging. J Knee Surg.

[9] Degnan AJ, Maldjian C, Adam RJ, Fu FH, Di Domenica M (2015). Comparison of Insall-Salvati ratios in children with an acute anterior cruciate ligament tear and a matched control population. AJR Am J Roentgenol.

[10] Lum ZC, Saiz AM, Pereira GC, Meehan JP (2020). Patella baja in total knee arthroplasty. J Am Acad Orthop Surg.

[11] Tischer T, Geier A, Lutter C, Enz A, Bader R, Kebbach M (2023). Patella height influences patellofemoral contact and kinematics following cruciate-retaining total knee replacement. J Orthop Res.

[12] Nishizawa Y, Matsumoto T, Kubo S, Muratsu H, Matsushita T, Oka S (2013). The influence of patella height on soft tissue balance in cruciate-retaining and posterior-stabilised total knee arthroplasty. Int Orthop.

[13] Portner O (2014). High tibial valgus osteotomy: closing, opening or combined? Patellar height as a determining factor. Clin Orthop Relat Res.

[14] Aglietti P, Buzzi R, D’andria S, Zaccherotti G. Patellofemoral problems after intraarticular anterior cruciate ligament reconstruction. Clin Orthop Relat Res. 1993;288195–204. 10.1097/00003086-199303000-00025. 8458134

[15] Igoumenou VG, Dimopoulos L, Mavrogenis AF (2019). Patellar height assessment methods: an update. JBJS Rev.

[16] Smith TO, Davies L, Toms AP, Hing CB, Donell ST (2011). The reliability and validity of radiological assessment for patellar instability. A systematic review and meta-analysis. Skelet Radiol.

[17] Chen HC, Lin CJ, Wu CH, Wang CK, Sun YN (2010). Automatic Insall-Salvati ratio measurement on lateral knee x-ray images using model-guided landmark localization. Phys Med Biol.

[18] Kim KC, Cho HC, Jang TJ, Choi JM, Seo JK (2021). Automatic detection and segmentation of lumbar vertebrae from X-ray images for compression fracture evaluation. Comput Methods Programs Biomed.

[19] Krogue JD, Cheng KV, Hwang KM, Toogood P, Meinberg EG, Geiger EJ (2020). Automatic hip fracture identification and functional subclassification with deep learning. Radiol Artif Intell.

[20] Qu Y, Li X, Yan Z, Zhao L, Zhang L, Liu C (2021). Surgical planning of pelvic tumor using multi-view CNN with relation-context representation learning. Med Image Anal.

[21] von Schacky CE, Wilhelm NJ, Schäfer VS, Leonhardt Y, Gassert FG, Foreman SC (2021). Multitask deep learning for segmentation and classification of primary bone tumors on radiographs. Radiology.

[22] Consalvo S, Hinterwimmer F, Neumann J, Steinborn M, Salzmann M, Seidl F (2022). Two-phase deep learning algorithm for detection and differentiation of ewing sarcoma and acute osteomyelitis in paediatric radiographs. Anticancer Res.

[23] Leung K, Zhang B, Tan J, Shen Y, Geras KJ, Babb JS (2020). Prediction of total knee replacement and diagnosis of osteoarthritis by using deep learning on knee radiographs: data from the osteoarthritis initiative. Radiology.

[24] Ye Q, Shen Q, Yang W, Huang S, Jiang Z, He L (2020). Development of automatic measurement for patellar height based on deep learning and knee radiographs. Eur Radiol.

[25] Kwolek K, Grzelecki D, Kwolek K, Marczak D, Kowalczewski J, Tyrakowski M (2023). Automated patellar height assessment on high-resolution radiographs with a novel deep learning-based approach. World J Orthop.

[26] Bonaldi L, Pretto A, Pirri C, Uccheddu F, Fontanella CG, Stecco C (2023). Deep learning-based medical images segmentation of Musculoskeletal anatomical structures: a survey of bottlenecks and strategies. Bioengineering.

[27] Sharbatdaran A, Romano D, Teichman K, Dev H, Raza SI, Goel A (2022). Deep learning automation of kidney, liver, and spleen segmentation for Organ volume measurements in autosomal Dominant polycystic kidney disease. Tomography.

[28] Papandreou G, Zhu T, Kanazawa N, Toshev A, Tompson J, Bregler C et al. Towards Accurate Multi-person Pose Estimation in the Wild; 2017. arXiv.org. Ithaca: Cornell University. Library. 10.1109/CVPR.2017.395.

[29] Lee J, Kim T, Beak S, Moon Y, Jeong J (2023). Real-time pose estimation based on ResNet-50 for rapid safety prevention and accident detection for field workers. Electronics.

[30] Li Y, Jia S, Li Q, BalanceHRNet (2023). An effective network for bottom-up human pose estimation. Neural Netw.

[31] Li Q, Zhang Z, Xiao F, Zhang F, Bhanu B. Dite-HRNet: Dynamic Lightweight High-Resolution Network for Human Pose Estimation; 2022. arXiv.org. Ithaca: Cornell University. Library. 10.24963/ijcai.2022/153.

[32] Cheng B, Xiao B, Wang J, Shi H, Huang TS, Zhang L, HigherHRNet. Scale-Aware Representation Learning for Bottom-Up Human Pose Estimation. In: Proceedings of the IEEE/CVF conference on computer vision and pattern recognition 2020 (pp. 5386–5395). 10.48550/arXiv.1908.10357.

[33] Wang J, Sun K, Cheng T, Jiang B, Deng C, Zhao Y (2021). Deep high-resolution representation learning for visual recognition. IEEE Trans Pattern Anal Mach Intell.

[34] Zou GY (2012). Sample size formulas for estimating intraclass correlation coefficients with precision and assurance. Stat Med.

